# Relationship between Chinese Baijiu consumption and dental caries among 55- to 74-year-old adults in Guangdong, southern China: a cross-sectional survey

**DOI:** 10.1186/s12877-021-02453-x

**Published:** 2021-09-25

**Authors:** Xiangyu Huang, Yihao Liang, Weihua Fan, Wei Liu, Buling Wu, Jianbo Li

**Affiliations:** 1 Stomatological Hospital, Southern Medical University, No. 366, South of Jiangnan Avenue, Guangzhou, 510280 China; 2 Faculty of School Health, Guangzhou Center for Disease Control and Prevention, Guangzhou, China; 3 Nanfang Hospital, Southern Medical University & Stomatological College of Southern Medical University, Guangzhou, 510080 China; 4 Shenzhen Stomatology Hospital (Pingshan), Southern Medical University, 143 Dongzong Road, Pingshan District, Shenzhen, 518118 China

**Keywords:** Alcohol consumption, Chinese Baijiu, Cross-sectional survey, Dental caries, Oral epidemiology

## Abstract

**Background:**

Whether an association between alcohol consumption and dental caries exists is still unclear. Chinese Baijiu is the most common alcohol consumed by middle-aged and elderly Chinese individuals. This study aimed to assess the relationship between alcohol consumption (Chinese Baijiu) and dental caries in Guangdong Province, southern China.

**Methods:**

A cross-sectional study was conducted in Guangdong Province using a multistage, stratified, equal-sized, random sampling strategy. In total, 576 individuals aged 55–74 were recruited to fill out a questionnaire through face-to-face and one-on-one interviews and to undergo a series of dental examinations with a Community Periodontal Index (CPI) probe. According to the standard for clinical dentition examination of the WHO 2013 criteria, the presence of dental caries was determined by the DFT/DFRoot (decayed-filled tooth/root) index. The ratios of males to females and urban people to countrymen were both 1:1. Then, the chi-square test and rank-sum tests were used to compare the differences in caries between subgroups, and multivariate logistic regression analyses, as well as negative binomial regression analyses, were executed to identify the potential relationship between alcohol consumption and caries.

**Results:**

The prevalence of crown caries was 79.17% with a DFT index of 3.19, while that of root caries was 61.28% with a DFRoot index of 2.08. The prevalence and mean tooth of crown caries of females were higher than those of males. The prevalence and mean DFRoot of root caries in rural areas were higher than those in urban areas. The results of the multivariate logistic regression analysis and negative binomial regression analysis showed that there was a statistically significant negative correlation between the consumption frequency of Chinese Baijiu and caries (often vs. never/rarely, crown caries: odds ratio (OR) = 0.54, 95% confidence interval (CI): 0.26–1.13, *P* = 0.103, incidence rate ratio (IRR) = 0.63, 95% CI: 0.44–0.92, *P* = 0.015; root caries: OR = 0.47, 95% CI: 0.24–0.93, *P* = 0.030, IRR = 0.52, 95% CI: 0.32–0.54, *P* = 0.008).

**Conclusions:**

Within the limitations of this study, frequent consumption of Chinese Baijiu was a protective factor for caries in middle-aged and elderly people in Guangdong Province. However, considering the harm of alcohol to one’s general health, it is recommended to drink moderately and avoid alcohol abuse.

**Supplementary Information:**

The online version contains supplementary material available at 10.1186/s12877-021-02453-x.

## Background

Dental caries is the most common chronic bacterial infectious disease and is an alarming public health problem worldwide. The prevalence of caries in permanent teeth ranks first among the 328 major diseases reported by the Lancet [1]. In 2015, approximately 2.5 billion people worldwide had untreated dental caries in their permanent teeth [2]. Untreated caries were found to be responsible for 12% of global productivity losses due to dental diseases [2]. Dental caries is one of the most common diseases seriously affecting oral health status. Oral health is an important part of general health.

According to the China Statistical Yearbook-2020 [3], the population aged 65 and over in China accounted for 12.6% of the total population, and the population aged 0–14 accounted for 16.8%, which means that China has entered an ageing society. With the gradual increase in middle-aged and elderly people, oral problems are gradually becoming prominent. With increasing numbers of patients retaining natural teeth into old age, the challenge of providing oral healthcare for the ageing population will undoubtedly increase. Among them, the high prevalence of dental caries has become one of the most common and most harmful diseases affecting the oral health-related quality of life of middle-aged and elderly people.

Globally, 48% of the adult population consumes alcohol [4]. In 2015, the estimated prevalence among the adult population was 18.4% for heavy episodic alcohol use (in the past 30 days) [5]. Alcohol use is the most common risk factor for oral diseases, such as oral cancer and periodontal disease [6]. However, the relationship between alcohol use and dental caries is inconsistent. Some studies indicated that alcohol consumption had a positive effect on dental caries [7–9], while some studies showed a negative correlation with alcohol consumption and caries [10, 11], and some studies showed no association between alcohol consumption and caries [12–14].

Due to cultural differences between China and foreign countries, the types of alcohol consumed by Chinese people and others are not very consistent. Chinese Baijiu, whose alcohol content is high, between 41% vol and 68% vol, is the most common alcohol consumed by middle-aged and elderly Chinese individuals. The most famous Chinese Baijiu, such as Kweichow Moutai, Wuliangye, Gujinggong Liquor, Jiannanchun Chiew, Luzhou Laojiao, Shanxi Fenjiu Liquor, Shaanxi Xifeng Liquor, and Guizhou Dongjiu, are all highly alcoholic spirits. According to the annual report data of listed companies, more than 98% of the eight most famous Chinese Baijiu were consumed in Mainland China. Chinese Baijiu refers to distilled spirits made from sorghum, wheat, corn, rice, or other grains using Daqu, Xiaoqu, Fuqu, or Mixed qu as sacchariferous and fermentative agents [15]. The brewing process of Chinese Baijiu includes cooking, saccharification, fermentation, distillation, ageing, and blending. Chinese Baijiu might affect dental caries by affecting the oral microenvironment, especially dental bacterial biofilms, due to its high alcohol content. The association between the consumption of Chinese Baijiu and dental caries was unclear. Therefore, this survey aimed to assess the relationship between the consumption of Chinese Baijiu and dental caries in Guangdong Province, southern China.

## Materials and methods

This cross-sectional study was conducted in Guangdong and was a part of the 4th National Oral Health Survey in China.

This survey was approved and revised by the Stomatological Ethics Committee of the Chinese Stomatological Association (Approval No.: 2014–003). Written informed consent was obtained from the participants before the study.

Briefly, aiming to determine the relationship between the consumption of Chinese Baijiu and dental caries, a total of 576 Guangdong residents aged 55 to 74 years were recruited to fill out a questionnaire and accept a series of dental examinations.

### Sample selection

Research participants were selected from individuals aged 55–74 living in Guangdong Province for more than 6 months. For an even age distribution sample, equal subjects were collected from two age groups, 55–64 years old and 65–74 years old. The sample size was estimated according to the N = deff μ $$ {\displaystyle \begin{array}{c}2\\ {}a/2\end{array}} $$ p(1-p)/δ^2^ formula. The estimated rate p was set to 74.17% based on the caries prevalence of 65–74-year-old subjects in the Guangdong in the Third National Oral Health Survey [16]. The two-sided test level α was set as 0.05, so the confidence of investigation μ was equal to 1.96 (here, 2.0 was used). The sampling design efficiency deff was set as 2. The acceptable error δ was set as 0.10 p. A sample size of 279 for each age group was calculated. However, the actual sample size for each age group was adjusted to 288 for the convenience of calculation and distribution.

After the final total sample size was 576, a four-stage, stratified, equal-sized, random sampling strategy was adopted to obtain a representative sample. In the first stage, 8 primary sampling units, including 4 counties and 4 districts, were randomly selected by using the probability proportionate to size (PPS) method. Sampling units were divided into the urban sample and the rural sample according to the area. Chancheng and Shunde of Foshan, Yuexiu of Guangzhou, and Jiangcheng of Yangjiang were chosen to represent the urban sample. Boluo of Huizhou, Lufeng of Shanwei, Raoping of Chaozhou, and Luoding of Yunfu were chosen to represent the rural samples. In the second stage, 3 streets or townships were extracted from each district or county by the PPS method. In the third stage, a total of 24 neighbourhood or village committees were included from all streets or townships by the PPS method. In the fourth stage, 24 research subjects (12 subjects in each sex and each age group) were determined by random quota sampling from each neighbourhood or village committee.

Persons with an oral disease requiring medication or even emergency treatment or with serious major systematic diseases or disadvantages who were unable or unwilling to complete the examination and questionnaire were excluded.

### Questionnaire and variables

Socio-demographic variables were obtained from an oral health paper questionnaire at a face-to-face and one-on-one interview by trained interviewers. Details are as follows: (1) regular information, including name, sex, and age. (2) Socio-economic status included registered permanent residence type, education level, and household income. (3) Personal lifestyle, including oral hygiene practices and attitudes, sweet consumption, smoking habits, and alcohol consumption.

Registered permanent residence type had two categories: rural and urban. The education level was divided into 3 categories by the years at school, including no more than 6 years (≤ 6 years, graduated from primary school or less), more than 6 but no more than 9 years (> 6, ≤9 years, graduated from primary school but no more than junior high school), and more than 9 years (> 9 years, higher than graduated from junior high school). Consumption of sweets was categorized by frequency into two levels: rarely/sometimes (less than once daily) and often (no less than once daily). The types of sweet intake included sweet snacks and sweet beverages. Smoking habits were registered in three categories according to cigarette usage: none, light (≤10 cigarettes daily), and heavy (> 10 cigarettes daily). Alcohol consumption, here specifically referring to Chinese Baijiu, was divided into three categories based on drinking frequency: never, sometimes (<once weekly), and often (≥once weekly). Household income (annual household income per capita) was classified into 3 levels by using quartiles: low (<Quartile 1), medium (Quartile 1 to Quartile 3), and high (> Quartile 3).

### Oral examination and measurement

The dental examination was carried out by three examiners and recorded by three assistants. All of the examiners were experienced dentists. Training and initial calibration trials for examiners were conducted by the technical team of the 4th National Oral Health Survey. The kappa values of the three examiners to the standard examiner were measured to exceed 0.8, which reflected great reliability for dental caries diagnosis in this study. Inter-examiner reproducibility was assessed by re-examination of approximately 5% of the participants during the examination. Inter-examiner kappa values of 0.88, 0.80, and 0.83 were obtained. The inter-examiner reproducibility was completely reliable.

The examination was conducted through visual and exploratory assessment. A consistent lightweight portable examination light (in the blue-white colour spectrum) was used as the light source in the examination. The plane front-surface dental mirror and CPI probe were used to inspect the caries state on the crown and root. All visible teeth were checked in a certain order. The tooth examination started from maxillary tooth #18 and continued to #28; then, the examination began again at mandible tooth #38 and continued to #48.

The diagnostic criteria of dental caries in this study referred to the standard of WHO 2013 guidelines [17] and are listed below:

1. No caries: no fillings due to caries, and a complete crown without signs of caries.
2. Crown caries (DT)/root caries (DRoot): crown or root with obvious cavities or under-enamel defect or soft-probing sensation.
3. Filled crown (FT)/filled root (FRoot): crown or root with permanent fillings (silver mercury, composite resin, glass ionomer, etc.) due to caries, but without any caries lesion. Filling involving both the crown and the root was recorded as FT and FRoot, respectively.

Prevalence was evaluated by the percentage of participants with a DFT/DFRoot score (> 0). The mean DFT/DFRoot was calculated to evaluate the severity of caries among the population.

### Statistical analyses

All survey data were double-entered into EpiData 3.0 (EpiData Association, Odense, Denmark) and validated to ensure accuracy. Chi-square tests and rank-sum tests were used to compare the differences in caries prevalence rate and the mean DFT/DFRoot between subgroups (e.g., sex, age, area) and for bivariate analysis with risk factor covariates (e.g., sweet consumption, smoking status). Multivariate logistic regression analyses and negative binomial regression models were applied to assess the relationship between alcohol consumption and caries prevalence rate or the mean DFT/DFRoot ratio, respectively. Odds ratios (ORs), incidence rate ratios (IRRs), and 95% confidence intervals (CIs) were estimated. Statistical analyses were conducted with SAS 9.4 software (SAS Institute, Inc., Carey, NC, USA). The test level α for statistical significance in the analyses was set to 0.05.

## Results

A total of 576 participants were included in this study: half were male and half were female, half were from urban areas and half were from rural areas, and half were aged 55–64 years old and half were aged 65–74 years old. Among all the subjects, 333 (57.81%) had less than 6 years at school, 135 (23.44%) had 6–9 years at school, and 108 (18.75%) had more than 9 years at school. This result reflected that more than half of the subjects had low education levels. A total of 458 (79.51%) subjects never or rarely consumed sweet food, while 118 (20.49%) consumed sweet food frequently. A total of 416 (72.22%) subjects declared they never smoked, 44 (7.64%) smoked less than 10 cigarettes per day, and 116 (20.14%) smoked more than 10 cigarettes per day. A total of 414 (71.86%) subjects never or rarely consumed alcohol (Chinese Baijiu), 113 (19.62%) sometimes consumed alcohol, and 49 (8.51%) frequently consumed alcohol. A total of 108 subjects had low-level household income, 293 had medium-level income, 118 had high-level income, and the other 57 subjects refused to disclose their income level.

The prevalence and mean tooth of dental caries among 55- to 74-year-old people in Guangdong Province are shown in Table 1. The status of dental caries in females was overall more serious than that in males. The prevalence of crown caries in females was 83.33%, which was significantly higher than the 75.00% prevalence in males (*P < 0.05*). Similarly, the mean DFT value in females was 3.68, while it was 2.71 in males (*P < 0.001*). In terms of root caries, there was no significant difference in sex in either prevalence or the mean DFRoot value. When comparing the status of caries between different age groups, the results showed that subjects in the 65–74 year group had both a higher prevalence and DTF or DFRoot value than those in the 55–64 year group (*P* > 0.05). The crown caries status was not significantly different between urban and rural subjects. However, the prevalence of root caries in rural areas was 66.67%, which was higher than the 55.90% prevalence in urban areas (*P < 0.01*), while the DFRoot of root caries in rural areas was 2.49%, which was higher than the 1.67% prevalence in urban areas (*P < 0.001*). Different caries situations were observed in subjects with different education levels. The prevalence and DTF or DFRoot value decreased along with the increase in the years at school (*P < 0.01*). Subjects who frequently consumed sweets had a higher crown caries prevalence than those who never or rarely consumed sweets (*P < 0.05*). The prevalence of crown caries varied irregularly with the frequency of smoking. The ≤10 cigarettes/day group had the lowest prevalence of 68.18%, while the never smoked group had the highest prevalence of 82.45% (*P < 0.01*). The DTF value significantly decreased from 3.36 to 3.12 and 2.69 in the three groups along with the increase in smoking frequency (*P < 0.001*). Interestingly, the prevalence of crown caries and root caries (*P < 0.05*), DTF value, and DFRoot value (*P < 0.01*) were significantly reduced with the growing frequency of alcohol consumption.

**Table 1:** The dental caries status among 55- to 74-year-old people in Guangdong Province

	N	Crown caries						Root caries					
		n	% ^a^	*P-value*	DFT	SD ^b^	*P-value*	n	% ^a^	*P-value*	DFRoot	SD ^b^	*P-value*
Total	576	456	79.17		3.19	3.26		353	61.28		2.08	2.78	
Sex				**0.014**			**< 0.001**			0.932			0.467
Male	288	216	75.00		2.71	2.97		177	61.46		1.94	2.58	
Female	288	240	83.33		3.68	3.46		176	61.11		2.22	2.96	
Age				0.218			0.053			0.347			0.055
55–64 years	288	222	77.08		2.94	3.13		171	59.38		1.82	2.49	
65–74 years	288	234	81.25		3.45	3.37		182	63.19		2.34	3.02	
Residence				0.305			0.907			**0.008**			**< 0.001**
Urban	288	233	80.90		3.15	3.17		161	55.90		1.67	2.56	
Rural	288	223	77.43		3.24	3.34		192	66.67		2.49	2.93	
Education level ^c^				0.241			**0.003**			**0.002**			**< 0.001**
≤ 6 years	333	271	81.38		3.57	3.40		218	65.47		2.48	3.07	
> 6, ≤9 years	135	105	77.78		2.97	3.34		85	62.96		1.81	2.43	
> 9 years	108	80	74.07		2.31	2.42		50	46.30		1.17	1.84	
Sweet consumption ^d^				**0.029**			0.067			0.569			0.582
Never/rarely	458	354	77.29		3.09	3.22		278	60.70		2.04	2.71	
Often	118	102	86.44		3.61	3.37		75	63.56		2.24	3.05	
Smoking status				**0.007**			**< 0.001**			0.679			0.227
Never smoked	416	343	82.45		3.51	3.36		259	62.26		2.20	2.85	
≤ 10 cigarettes/day	44	30	68.18		2.66	3.12		27	61.36		2.09	3.13	
> 10 cigarettes/day	116	83	71.55		2.25	2.69		67	57.76		1.63	2.31	
Alcohol consumption ^e^				**0.019**			**0.002**			**0.039**			**0.008**
Never/rarely	414	338	81.64		3.42	3.37		263	63.53		2.21	2.88	
Sometimes	113	86	76.11		3.04	3.15		68	60.18		2.06	2.76	
Often	49	32	65.31		1.69	1.81		22	44.90		0.96	1.41	
Household income ^f^				0.422			0.309			0.222			**0.027**
Low	108	82	75.93		3.30	3.59		66	61.11		2.41	3.07	
Medium	293	229	78.16		3.02	3.15		186	63.48		1.94	2.60	
High	118	96	81.36		3.28	3.21		63	53.39		1.69	2.63	
Refuse to disclose	57	49	85.96		3.72	3.26		38	66.67		2.95	3.18	

a. %: equals to n/N*100

b. SD: Standard deviation

c. Education level was divided by years at school. 6 years: primary school, 9 years: junior high school, > 9 years: more than junior high school.

d. Sweet consumption: rarely/sometimes means less than once daily; often means once daily or more.

e. Alcohol consumption (Chinese Baijiu): never; sometimes means less than once weekly; often means once weekly or more.

f. Household income was classified by quartiles. Low means within quartile 1, medium means from quartile 1 to quartile 3, and high means beyond quartile 3.

The results of the multivariate logistic regression analysis and the negative binomial regression analysis are shown in Tables 2 and 3. After controlling for the influence of confounding factors such as sex, age, residence, education level, smoking status, and household income, the results showed that the influence of Chinese Baijiu consumption on the prevalence of crown caries was not statistically significant (*P* > 0.05) but a statistically significant negative correlation with root caries prevalence (OR = 0.47, *P < 0.05*), mean DFT (IRR = 0.63, *P = 0.015*) and mean DFRoot (IRR = 0.52, *P = 0.008*).

**Table 2:** The relationship between the prevalence of caries and alcohol consumption (results of the multivariate logistic regression analyses)

	Crown caries			Root caries		
	*OR* (95% *CI*) ^#^	Adjusted *OR* (95% *CI*)	*P-value*	*OR* (95% *CI*) ^#^	Adjusted *OR* (95% *CI*)	*P-value*
Alcohol consumption						
Never/ rarely	ref.	ref.		ref.	ref.	
Sometimes	0.72 (0.44, 1.18)	0.74 (0.42, 1.29)	0.285	0.87 (0.57, 1.33)	0.83 (0.51, 1.36)	0.458
Often	0.42 (0.22, 0.80)	0.54 (0.26, 1.13)	0.103	0.47 (0.26, 0.85)	**0.47 (0.24, 0.93)**	**0.030**
Sex						
Male	ref.	ref.		ref.	ref.	
Female	1.67 (1.11, 2.51)	1.08 (0.61, 1.93)	0.786	0.99 (0.70, 1.38)	0.62 (0.38, 1.01)	0.055
Age						
55–64 years	ref.	ref.		ref.	ref.	
65–74 years	1.29 (0.86, 1.93)	1.08 (0.69, 1.70)	0.724	1.17 (0.84, 1.64)	0.93 (0.64, 1.36)	0.718
Residence						
Urban	ref.	ref.		ref.	ref.	
Rural	0.81 (0.54, 1.21)	1.06 (0.63, 1.77)	0.828	1.58 (1.13, 2.21)	1.53 (0.98, 2.37)	0.059
Education level						
≤ 6 years	ref.	ref.		ref.	ref.	
> 6, ≤ 9 years	0.80 (0.49, 1.31)	0.98 (0.56, 1.73)	0.953	0.90 (0.59, 1.36)	0.97 (0.60, 1.57)	0.894
> 9 years	0.65 (0.39, 1.09)	0.63 (0.35, 1.14)	0.127	0.45 (0.29, 0.71)	**0.45 (0.27, 0.76)**	**0.002**
Sweet consumption						
Never/ rarely	ref.	ref.		ref.	ref.	
Often	1.87 (1.06, 3.31)	**1.85 (1.00, 3.40)**	**0.049**	1.13 (0.74, 1.72)	1.55 (0.96, 2.49)	0.073
Smoking status						
Never smoked	ref.	ref.		ref.	ref.	
≤ 10 /day	0.46 (0.23, 0.90)	0.56 (0.26, 1.25)	0.157	0.96 (0.51, 1.82)	0.82 (0.39, 1.71)	0.599
> 10 /day	0.54 (0.33, 0.86)	0.66 (0.35, 1.26)	0.206	0.83 (0.55, 1.26)	0.62 (0.35, 1.11)	0.108
Household income						
Low	ref.	ref.		ref.	ref.	
Medium	1.14 (0.67, 1.91)	1.13 (0.65, 1.97)	0.659	1.11 (0.70, 1.74)	1.22 (0.76, 1.98)	0.415
High	1.38 (0.73, 2.62)	1.41 (0.67, 2.97)	0.363	0.73 (0.43, 1.24)	1.00 (0.54, 1.84)	0.992

#: OR, odds ratio

**Table 3:** The relationship between the mean DFT/DFRoot of caries and alcohol consumption (results of the negative binomial regression analyses)

	DFT			DFRoot		
	*IRR* (95% CI) ^※^	Adjusted *IRR* (95% CI)	*P-value*	*IRR* (95% CI) ^※^	Adjusted *IRR* (95% CI)	*P-value*
Alcohol consumption						
Never/ rarely	ref.	ref.		ref.	ref.	
Sometimes	0.89 (0.72, 1.10)	1.06 (0.83, 1.35)	0.646	0.93 (0.70, 1.24)	1.18 (0.87, 1.61)	0.287
Often	0.50 (0.35, 0.70)	**0.63 (0.44, 0.92)**	**0.015**	0.43 (0.27, 0.68)	**0.52 (0.32, 0.84)**	**0.008**
Sex						
Male	ref.	ref.		ref.	ref.	
Female	1.36 (1.14, 1.61)	1.07 (0.85, 1.35)	0.550	1.14 (0.91, 1.43)	0.83 (0.62, 1.12)	0.232
Age						
55–64 years	ref.	ref.		ref.	ref.	
65–74 years	1.17 (0.99, 1.39)	1.04 (0.87, 1.25)	0.650	1.29 (1.03, 1.61)	1.02 (0.80, 1.29)	0.874
Residence						
Urban	ref.	ref.		ref.	ref.	
Rural	1.03 (0.86, 1.22)	1.11 (0.90, 1.38)	0.341	1.49 (1.19, 1.86)	**1.59 (1.20, 2.10)**	**0.001**
Education level						
≤ 6 years	ref.	ref.		ref.	ref.	
> 6, ≤9 years	0.83 (0.68, 1.02)	0.91 (0.72, 1.15)	0.417	0.73 (0.55, 0.95)	0.78 (0.57, 1.05)	0.104
> 9 years	0.65 (0.51, 0.81)	**0.66 (0.51, 0.86)**	**0.002**	0.47 (0.34, 0.64)	**0.50 (0.35, 0.71)**	**< 0.001**
Sweet consumption						
Never/ rarely	ref.	ref.		ref.	ref.	
Often	1.17 (0.95, 1.44)	**1.30 (1.04, 1.63)**	**0.023**	1.10 (0.83, 1.45)	**1.32 (1.14, 2.06)**	**0.005**
Smoking status						
Never smoked	ref.	ref.		ref.	ref.	
≤ 10 /day	0.76 (0.54, 1.05)	0.82 (0.57, 1.18)	0.281	0.95 (0.62, 1.46)	0.90 (0.57, 1.42)	0.643
> 10 /day	0.64 (0.51, 0.80)	**0.72 (0.54, 0.96)**	**0.027**	0.74 (0.55, 0.99)	0.71 (0.49, 1.03)	0.070
Household income						
Low	ref.	ref.		ref.	ref.	
Medium	0.92 (0.73, 1.16)	0.93 (0.74, 1.18)	0.561	0.81 (0.60, 1.09)	0.86 (0.64, 1.15)	0.307
High	0.99 (0.75, 1.31)	1.06 (0.78, 1.44)	0.700	0.70 (0.49, 1.01)	0.97 (0.66, 1.44)	0.883

※: IRR, incidence rate ratio

In addition, after controlling for the influence of confounding factors, rural residence was a risk factor for the mean DFRoot value (IRR = 1.59, *P = 0.001*). The education level was significantly negatively correlated with the mean DFT value (IRR = 0.66, *P = 0.002*), mean DFRoot value (IRR = 0.50, *P < 0.001*), and root caries prevalence (OR = 0.45, *P = 0.002*). Sweet consumption was significantly positively correlated with crown caries prevalence (OR = 1.85, *P < 0.05*), the mean DFT value (IRR = 1.30, *P = 0.023*), and the mean DFRoot value (IRR = 1.32, *P = 0.005*). Smoking was significantly negatively associated with the mean DFT value (IRR = 0.72, *P = 0.027*).

## Discussion

The oral health of elderly adults was closely related to their living habits and affected their physical and mental condition. Dental caries was one of the most common oral diseases. Therefore, it is a problem worthy of concern.

In the current study, the status of dental caries among 55- to 74-year-old adults in southern China was investigated. The results showed that the prevalence of dental caries was 79.7%, with a mean DFT of 3.19, which was higher than that of elderly individuals in northeastern China (prevalence 67.5%, DFT 2.68) [18].

Several factors were found to be related to dental caries in elderly individuals. Frequent alcohol consumption and heavy smoking were protective factors, while rural living, low education level and frequent sweet consumption were risk factors.

Alcohol use is a leading risk factor for death and disability, but its overall association with health remains complex given the possible protective effects of moderate alcohol consumption on some conditions [19]. As this study aimed to identify the relationship between the consumption of Chinese Baijiu and dental caries with the ultimate goal of helping to identify whether alcohol control was suitable for caries prevention strategies after controlling for the influence of confounding factors (e.g., sex, age, residence, smoking status, household income, etc.), we were surprised to find that there was a statistically significant negative correlation between the consumption of Chinese Baijiu and caries. Consistently, in the study of Dutch in 2013, a weak but significant negative correlation between alcohol and enamel caries was found among 18- to 32-year-old adults [11]. In contrast, some studies drew different conclusions. A longitudinal investigation from 1979 to 1990 in Stockholm revealed that individuals with high alcohol consumption had significantly more surface decay and apical lesions [8]. Studies in Northern Europe found that individuals with high alcohol consumption had significantly more decayed teeth [7–9]. However, studies in Southern Europe [12], Western Europe [13], Central Europe [14], North America [20], and sub-Saharan Africa [21] found that there was no relation between alcohol consumption and caries.

How alcohol affects caries is not clear. The relationship between alcohol consumption and lifestyle and health is complex and multidimensional. Alcohol use affects caries through direct biological effects and indirect life-behaviour changes [22]. Alcohol exerts its direct biological effect by changing the salivary flow rate, salivary pH values, and bacterial species richness of sub- and supragingival plaques, as well as disturbing the host defence system [14]. A previous study indicated that moderate alcohol intake can stimulate the salivary secretion of parotid saliva [23]. Mutant streptococci (MS) is the main pathogen of caries. The decrease in oral colonization of MS was significantly related to the duration and quantity of alcohol use [24].

Moreover, oral hygiene and dental care habits modified by alcohol consumption also indirectly affect the occurrence of dental caries [8]. Different kinds of alcoholic beverages with different components may have different effects on the oral environment, which may lead to the inconsistent results mentioned above. Compared to beer, wine, and cocktails, Chinese Baijiu was considered a highly alcoholic spirit, with a higher ethanol concentration and lower sugar content. The impact of Chinese Baijiu on the oral environment was more similar to medical alcohol. Chinese Baijiu might affect dental caries by affecting the oral microenvironment, especially dental bacterial biofilms, due to the high alcohol content.

Alcohol is regarded to be detrimentally associated with the occurrence of many chronic diseases, including oral cancer, liver cancer, breast cancer, hypertensive disease, and cirrhosis of the liver. However, the influence of alcohol on health is related not only to the average alcohol consumption but also to the drinking pattern [22]. Regular light or moderate drinking was found to be a protective factor against coronary heart disease [25]. Chinese Baijiu was reported to have the benefit of coronary artery disease therapy by increasing nitric oxide bioactivity [26].

In addition to alcohol consumption, heavy smokers had lower DFT values. Smoking is a risk factor for periodontal disease. However, it is not clear whether smoking affects crown caries. Further research is needed.

Low education level, rural living, and frequent sweet consumption were risk factors for caries.

A study in Finland showed that a basic level of education was strongly associated with the occurrence of dental caries [27]. In a Greece study, educational level was analysed to be the only predictor of DMFS in senior citizens [28]. Root Caries Index scores were higher among elderly individuals in rural areas than among those in urban areas in the middle of China [29]. Low education level or rural living indicated low socioeconomic status in China. A systematic review showed that populations with the highest proportions of people with low socioeconomic status are associated with a greater severity of caries [30]. The results of this study are consistent with previous studies.

As reports mentioned, people with nine or more sweets consumed per day had double odds of obtaining root caries [31]. Sweet consumption was found to be a risk factor for both crown and root caries, which coincided with common knowledge.

The limitation of this survey was that the DFT index was less accurate than the DFS index, as a tooth has multiple tooth surfaces and caries typically involve multiple tooth surfaces rather than one. Studies using the DFS index are indicated. Bring more types of alcoholic beverages into the study and distinguishing the alcohol consumption pattern could help better understand the relationship between alcohol consumption and caries.

In general, our study reflects the overview of dental caries in middle-aged and elderly people in Guangdong Province and its relationship with Chinese Baijiu consumption. It provides an epidemiological basis for caries prevention in middle-aged and elderly people. Moreover, the underlying mechanism of alcohol consumption in root caries reduction needs to be further explored.

## Conclusions

Knowledge about the influence of alcohol on caries is inadequate. In this survey, the consumption of Chinese Baijiu was a protective factor against caries in middle-aged and elderly people in Guangdong Province. However, considering the harm that alcohol causes to one’s general health, it is recommended to drink moderately and avoid alcohol abuse.

## Supplementary Information

**Additional file 1: Table 1.** The prevalence of crown caries among the population aged 55–74 years old in Guangdong Province. **Table 2.** The mean DFT of crown caries among the population aged 55–74 years old in Guangdong Province. **Table 3.** The prevalence of root caries among the population aged 55–74 years old in Guangdong Province. **Table 4.** The mean DFRoot of root caries among the population aged 55–74 years old in Guangdong Province.

## Data Availability

The data sets used and/or analyzed during the current study are available from the corresponding author on reasonable request.

## Abbreviations

CI(s): Confidence Interval(s)
CPI: Community Periodontal Index
DT: Decayed Tooth (Crown)
DRoot: Decayed Root
FT: Filled Tooth (Crown)
FRoot: Filled Root
DFRoot: Decayed, Filled (permanent) Root
D(M)FS: Decayed, (Missing), Filled (permanent tooth crown) Surface
DFT: Decayed, Filled (permanent) Tooth (Crown)
IRR(s): Incidence Rate Ratio(s)
MS: mutans streptococci
OR(s): Odds Ratio(s)
PPS: Probability Proportional to Size
SD: Standard deviation
