# Supplementary material for: Relationship between Chinese Baijiu consumption and dental caries among 55- to 74-year-old adults in Guangdong, southern China: a cross-sectional survey
Source: BMC Geriatr. 2021 Sep 25;21:506. doi: 10.1186/s12877-021-02453-x (PMC8466654; doi:10.1186/s12877-021-02453-x)
Supplement: Supplementary file 1 — Additional file 1: Table 1. The prevalence of crown caries among the population aged 55–74 years old in Guangdong Province. Table 2. The mean DFT of crown caries among the population aged 55–74 years old in Guangdong Province. Table 3. The prevalence of root caries among the population aged 55–74 years old in Guangdong Province. Table 4. The mean DFRoot of root caries among the population aged 55–74 years old in Guangdong Province. [file 12877_2021_2453_MOESM1_ESM.docx]

Table 1 The prevalence of crown caries among the population aged 55-74 years old in Guangdong Province

| Age | Region/Sex | N | D | |  | F | |  | DF | | P |
| --- | --- | --- | --- | --- | --- | --- | --- | --- | --- | --- | --- |
|  |  |  | n | % |  | n | % |  | n | % |  |
| 55-64 years | Urban | 144 | 90 | 62.50 |  | 65 | 45.14 |  | 113 | 78.47 | 0.575* |
|  | Rural | 144 | 105 | 72.92 |  | 19 | 13.19 |  | 109 | 75.69 |  |
|  | Male | 144 | 91 | 63.19 |  | 32 | 22.22 |  | 101 | 70.14 | 0.005** |
|  | Female | 144 | 104 | 72.22 |  | 52 | 36.11 |  | 121 | 84.03 |  |
|  | Subtotal | 288 | 195 | 67.71 |  | 84 | 29.17 |  | 222 | 77.08 |  |
| 65-74 years | Urban | 144 | 97 | 67.36 |  | 60 | 41.67 |  | 120 | 83.33 | 0.365* |
|  | Rural | 144 | 112 | 77.78 |  | 10 | 6.94 |  | 114 | 79.17 |  |
|  | Male | 144 | 104 | 72.22 |  | 29 | 20.14 |  | 115 | 79.86 | 0.546** |
|  | Female | 144 | 105 | 72.92 |  | 41 | 28.47 |  | 119 | 82.64 |  |
|  | Subtotal | 288 | 209 | 72.57 |  | 70 | 24.31 |  | 234 | 81.25 |  |
| Total |  | 576 | 404 | 70.14 |  | 154 | 26.74 |  | 456 | 79.17 |  |

*P values for comparison between urban and rural areas, **P values for comparison between males and females.

Table 2 The mean DFT of crown caries among the population aged 55-74 years old in Guangdong Province

| Age | Region/Sex | N | DT | |  | FT | |  | DFT | | P |
| --- | --- | --- | --- | --- | --- | --- | --- | --- | --- | --- | --- |
|  |  |  | Number of teeth | Mean±SD |  | Number of teeth | Mean±SD |  | Number of teeth | Mean±SD |  |
| 55-64 years | Urban | 144 | 261 | 1.81±2.44 |  | 156 | 1.08±1.67 |  | 417 | 2.90±3.08 | 0.821* |
|  | Rural | 144 | 400 | 2.78±3.06 |  | 29 | 0.20±0.62 |  | 429 | 2.98±3.18 |  |
|  | Male | 144 | 303 | 2.10±2.59 |  | 51 | 0.35±0.75 |  | 354 | 2.46±2.67 | 0.009** |
|  | Female | 144 | 358 | 2.49±3.01 |  | 134 | 0.93±1.68 |  | 492 | 3.42±3.47 |  |
|  | Subtotal | 288 | 661 | 2.30±2.81 |  | 185 | 0.64±1.33 |  | 846 | 2.94±3.13 |  |
| 65-74 years | Urban | 144 | 344 | 2.39±2.97 |  | 147 | 1.02±1.66 |  | 491 | 3.41±3.25 | 0.834* |
|  | Rural | 144 | 487 | 3.38±3.50 |  | 16 | 0.11±0.49 |  | 503 | 3.49±3.49 |  |
|  | Male | 144 | 371 | 2.58±3.19 |  | 56 | 0.39±0.99 |  | 427 | 2.97±3.24 | 0.014** |
|  | Female | 144 | 460 | 3.19±3.34 |  | 107 | 0.74±1.54 |  | 567 | 3.94±3.44 |  |
|  | Subtotal | 288 | 831 | 2.89±3.28 |  | 163 | 0.57±1.30 |  | 994 | 3.45±3.37 |  |
| Total |  | 576 | 1492 |  |  | 348 |  |  | 1840 |  |  |

*P values for comparison between urban and rural areas, **P values for comparison between males and females.

Table 3 The prevalence of root caries among the population aged 55-74 years old in Guangdong Province

| Age | Region/Sex | N | D | |  | F | |  | DF | | P |
| --- | --- | --- | --- | --- | --- | --- | --- | --- | --- | --- | --- |
|  |  |  | n | % |  | n | % |  | n | % |  |
| 55-64 years | Urban | 144 | 71 | 49.31 |  | 17 | 11.81 |  | 79 | 54.86 | 0.119* |
|  | Rural | 144 | 91 | 63.24 |  | 2 | 1.39 |  | 92 | 63.89 |  |
|  | Male | 144 | 83 | 57.64 |  | 9 | 6.25 |  | 88 | 61.11 | 0.549** |
|  | Female | 144 | 79 | 54.86 |  | 10 | 6.94 |  | 83 | 57.64 |  |
|  | Subtotal | 288 | 162 | 56.25 |  | 19 | 6.60 |  | 171 | 59.38 |  |
| 65-74 years | Urban | 144 | 80 | 55.56 |  | 9 | 6.25 |  | 82 | 56.94 | 0.028* |
|  | Rural | 144 | 98 | 68.06 |  | 7 | 4.86 |  | 100 | 69.44 |  |
|  | Male | 144 | 87 | 60.42 |  | 9 | 6.25 |  | 89 | 61.81 | 0.625** |
|  | Female | 144 | 91 | 63.19 |  | 7 | 4.86 |  | 93 | 64.58 |  |
|  | Subtotal | 288 | 178 | 61.81 |  | 16 | 5.56 |  | 120 | 63.19 |  |
| Total |  | 576 | 340 | 59.03 |  | 35 | 6.08 |  | 291 | 50.52 |  |

*P values for comparison between urban and rural areas, **P values for comparison between males and females.

Table 4 The mean DFRoot of root caries among the population aged 55-74 years old in Guangdong Province

| Age | Region/Sex | N | DRoot | |  | | FRoot | |  | DFRoot | | P |
| --- | --- | --- | --- | --- | --- | --- | --- | --- | --- | --- | --- | --- |
|  |  |  | Number of root | Mean±SD |  | Number of root | | Mean±SD |  | Number of root | Mean±SD |  |
| 55-64 years | Urban | 144 | 171 | 1.19±1.88 |  | 46 | | 0.04±0.45 |  | 217 | 1.51±2.25 | 0.035* |
|  | Rural | 144 | 304 | 2.11±2.67 |  | 2 | | 0.01±0.12 |  | 306 | 2.13±2.68 |  |
|  | Male | 144 | 243 | 1.69±2.30 |  | 16 | | 0.11±0.50 |  | 259 | 1.80±2.37 | 0.906** |
|  | Female | 144 | 232 | 1.61±2.41 |  | 32 | | 0.22±1.01 |  | 264 | 1.83±2.61 |  |
|  | Subtotal | 288 | 475 | 1.65±2.35 |  | 48 | | 0.17±0.80 |  | 523 | 1.82±2.49 |  |
| 65-74 years | Urban | 144 | 239 | 1.66±2.58 |  | 25 | | 0.17±0.86 |  | 264 | 1.83±2.83 | 0.004* |
|  | Rural | 144 | 393 | 2.73±3.11 |  | 17 | | 0.12±0.62 |  | 410 | 2.85±3.13 |  |
|  | Male | 144 | 276 | 1.92±2.65 |  | 24 | | 0.17±0.78 |  | 300 | 2.08±2.77 | 0.149** |
|  | Female | 144 | 356 | 2.47±3.12 |  | 18 | | 0.13±0.72 |  | 374 | 2.60±3.24 |  |
|  | Subtotal | 288 | 632 | 2.19±2.90 |  | 42 | | 0.15±0.75 |  | 674 | 2.34±3.02 |  |
| Total |  | 576 | 1107 |  |  | 90 | |  |  | 1197 |  |  |

*P values for comparison between urban and rural areas, **P values for comparison between males and females.
